# Report of the Board of Health of the City of Chicago, for 1867, 1868, and 1869, and a Sanitary History of Chicago, from 1833 to 1870

**Published:** 1871-06

**Authors:** 


Report of the Board of Health of the city of Chicago, for 1867, 1868, and 1869, and a Sanitary History of Chicago, from 1833 to 1870. Chicago: Lakeside Publishing and Printing Company.

We acknowledge the receipt of a handsomely bound copy of this valuable Report.

